# 2A and 2A-like Sequences: Distribution in Different Virus Species and Applications in Biotechnology

**DOI:** 10.3390/v13112160

**Published:** 2021-10-26

**Authors:** Juliana G. S. de Lima, Daniel C. F. Lanza

**Affiliations:** 1 Applied Molecular Biology Lab—LAPLIC, Department of Biochemistry, Federal University of Rio Grande do Norte, Natal 59064-720, Brazil; julianagabriela91@gmail.com; 2 Postgraduate Program in Biochemistry, Federal University of Rio Grande do Norte, Natal 59064-720, Brazil

**Keywords:** 2A peptide, double-stranded RNA virus, positive-sense single-stranded RNA virus, Totiviridae, Picornaviridae

## Abstract

2A is an oligopeptide sequence that mediates a ribosome “skipping” effect and can mediate a co-translation cleavage of polyproteins. These sequences are widely distributed from insect to mammalian viruses and could act by accelerating adaptive capacity. These sequences have been used in many heterologous co-expression systems because they are versatile tools for cleaving proteins of biotechnological interest. In this work, we review and update the occurrence of 2A/2A-like sequences in different groups of viruses by screening the sequences available in the National Center for Biotechnology Information database. Interestingly, we reported the occurrence of 2A-like for the first time in 69 sequences. Among these, 62 corresponded to positive single-stranded RNA species, six to double stranded RNA viruses, and one to a negative-sense single-stranded RNA virus. The importance of these sequences for viral evolution and their potential in biotechnological applications are also discussed.

## 1. Introduction

2A and 2A-like sequences are oligopeptides with approximately 18–25 amino acids and can mediate a co-translation “cleavage” of polyproteins in eukaryotic cells. The “core” sequence at the C-terminus of 2A, together with the N-terminal proline of the downstream protein, contains the canonical motif—(G/H)_1_D_2_(V/I)_3_E_4_X_5_N_6_P_7_G_8_P_9_—involved in a ribosome “skipping” effect during translation, which separates two proteins without needing a proteinase [1,2].

The 2A cleavage occurs between the G_8_ site at the upstream protein (P1) and the P_9_ site at the downstream protein (P2). During amino acid insertion into the protein, the 2A sequence can cause a structural modification at the ribosome peptidyl-transferase center (PTC), making the ribosome “skip” the proline codon. It inhibits the formation of a glycine-proline peptide bond because of the hydrolysis of the peptidyl (2A)-tRNAGly ester linkage, releasing the polypeptide from the translational complex [3,4]. In this way, the first amino acid, proline, of the downstream encoded protein, is specified by the third codon in the sequence of P_7_G_8_P_9_, and the C-terminal amino acid of the upstream encoded protein is a glycine encoded by the second codon in that sequence [5,6]. This ribosome “skipping” effect is also referred to as “Stop-Carry On” or “StopGo” translation [6]. Thus, the ribosome activity does not depend on structural elements within the mRNA but a peptide sequence, differentiating this mechanism from the other forms of non-canonical mRNA processing. Because of this activity, the 2A and 2A-like sequences can be named CHYSELs (cis-acting hydrolase elements) [7].

Originally, the term “2A” was assigned to define a specific region of the genome of the foot-and-mouth disease virus (FMDV), a positive-sense single-stranded RNA (pssRNA) virus and member of the Picornaviridae family [1,4,8,9,10]. Similar sequences discovered in other viruses were named “2A-like.” These sequences have been described in other Picornaviridae, such as *Equine rhinitis A* virus and *Porcine teschovirus-1*, in other viruses of the Dicistroviridae and Iflaviviridae families [2], and even in the infectious myonecrosis virus (IMNV), a double-stranded RNA (dsRNA) virus belonging to the Totiviridae family [11].

From these first discoveries, the 2A and 2A-like proteolytic cleavage activities have been demonstrated in several eukaryotic systems in vitro and in vivo [2,12]. Because of their mechanism of action, some authors also refer to 2A and 2A-like peptides as cis-acting hydrolase elements [7,13].

In 2017, Yang et al. reviewed the 2A sequence structures and functions of Picornaviridae members [14]. The latest works analyzing 2A and 2A-like sequences, including viruses from other families, were conducted by Luke et al. in 2008, 2009, and 2014 and by Luke and Ryan in 2013 [2,15,16,17]. With advances in sequencing technology, in recent years, there has been a significant increase in the number of viral sequences added to the National Center for Biotechnology Information (NCBI) database. Therefore, the goal of this article was to introduce a new screening of 2A and 2A-like sequences in viral genomes available from the NCBI database to revise the principal 2A and 2A-like sequences, describe their occurrence in different viral families, and discuss their potential applications in biotechnology.

## 2. Materials and Methods

The sequences used in this study were obtained from the viral databank (https://www.ncbi.nlm.nih.gov/genome/viruses/, accessed on 9 January 2021). To find 2A/2A-like sequences, the viral genomes were aligned against some of the 2A/2A-like classical motifs (GDVEENPGP; GDVESNPGP; HDIETNPGP; GDVELNPGP; GDIELNPGP; GDIESNPGP; HDVEMNPGP) using the Blastp tool (https://blast.ncbi.nlm.nih.gov/Blast.cgi, accessed on 9 January 2021) and the non-redundant protein sequences database (nr) only including viruses (taxid:10239). Search parameters were set to return a maximum of 500 sequences for each query. Repeated viral sequences were excluded from the analysis.

An active search was performed on the publication linked to the sequence annotation in the NCBI database to identify whether the sequences found had already been reported in the literature after the initial screening. If no report was found, an active search was performed using the Google Scholar search tool, with each respective virus name plus the word “2A” as keywords. If no articles reported the presence of 2A/2A-like in the query virus, we considered this finding novel.

## 3. Results and Discussion

### 3.1. 2A/2A-Like Distribution on Viruses

Table 1 shows the principal 2A or 2A-like motifs that had their self-cleavage efficiencies tested in vitro, confirming that these sequences are widely distributed among the pssRNA and dsRNA viruses, ranging from insect to mammalian viruses. Luke et al. were the first to report this wide distribution and identified motifs similar to those found in the FMDV [2].

The search for these motifs in the viral genomes available in the NCBI database revealed 69 sequences containing 2A-like motifs that had not been identified. Among these, 62 corresponded to pssRNA, six to dsRNA, and one to a negative-sense single-stranded RNA (nssRNA) virus. Additionally, 2A-like motifs, previously described in 102 sequences, were confirmed. All 2A/2A-like motifs and their respective species resulting from the search are described in Table 2 and Table 3.

### 3.2. pssRNA Viruses

**Figure 1:**
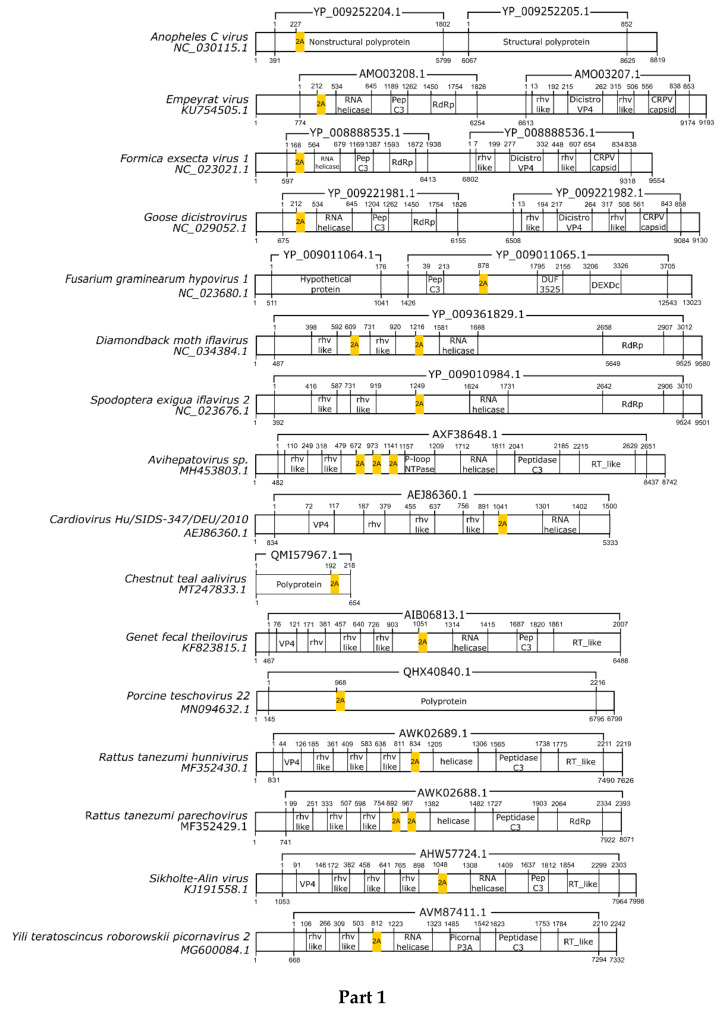

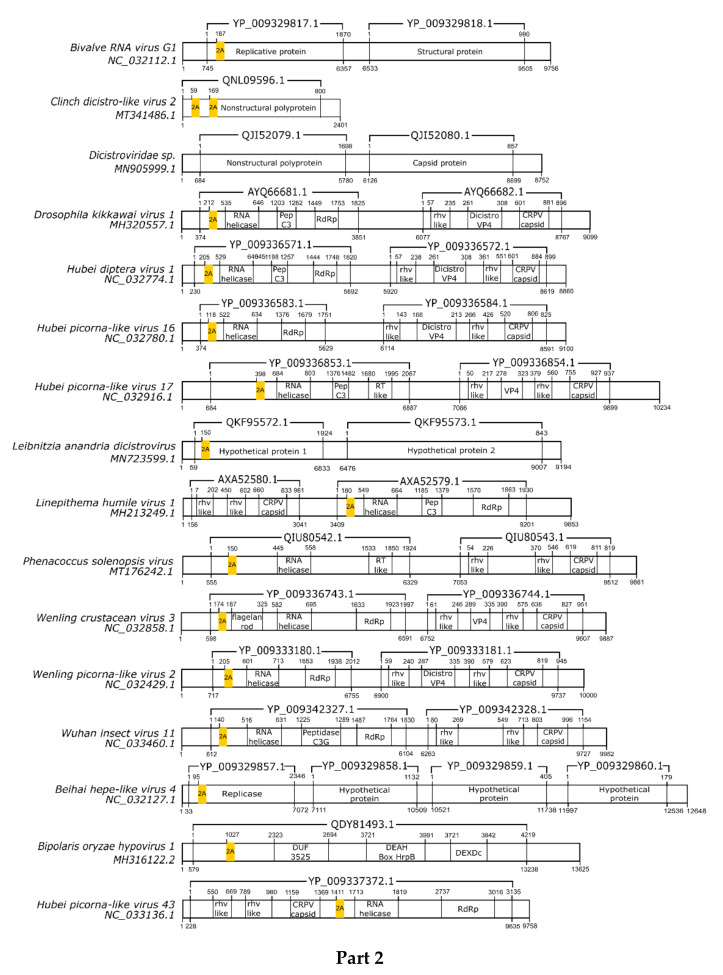

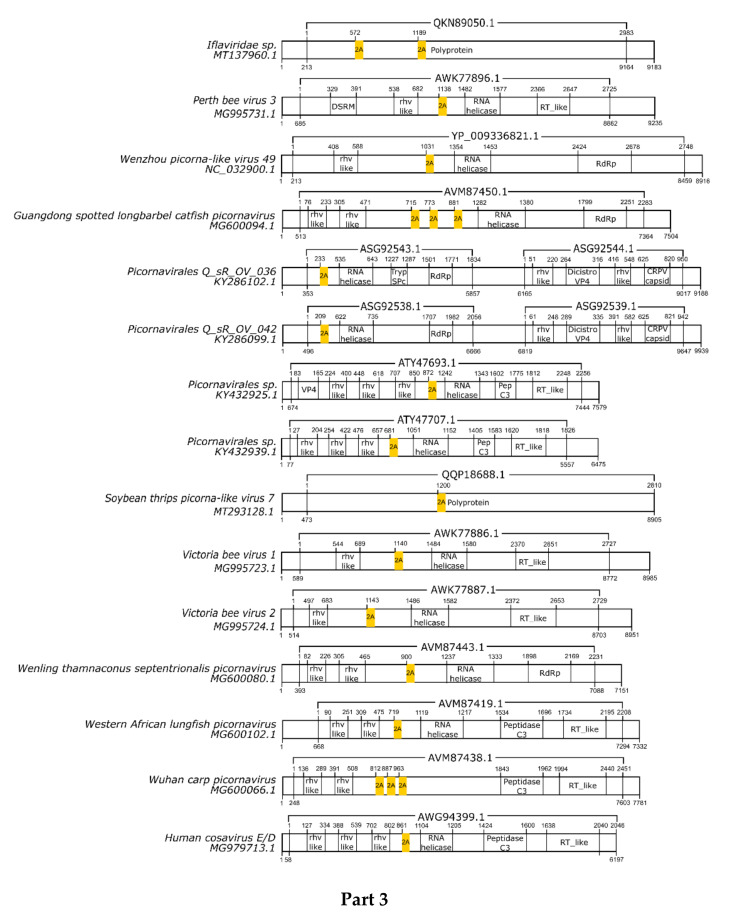

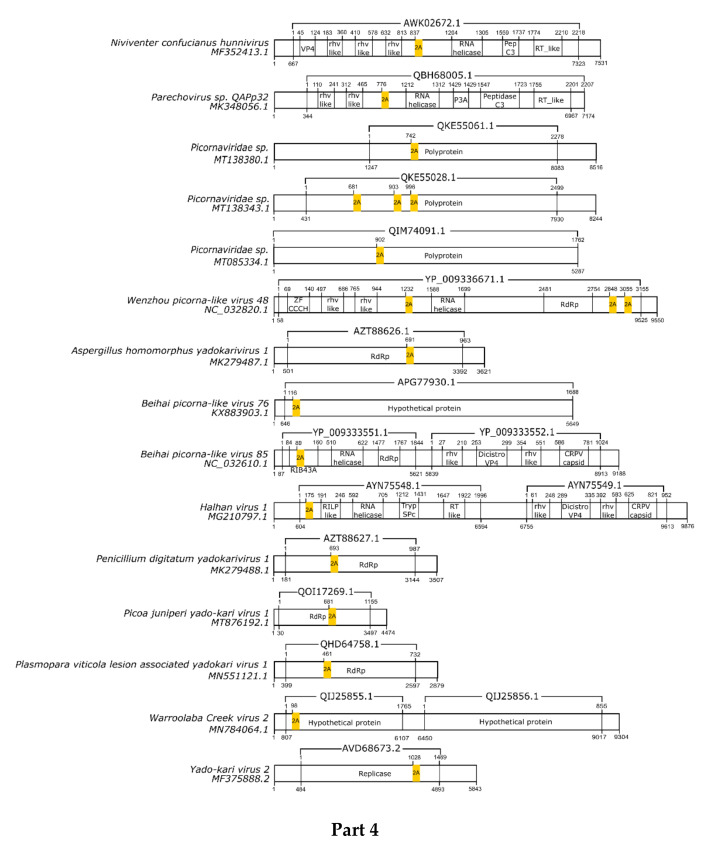
Schematic representation of positive-sense single-strand RNA virus sequences. Schematic representations of pssRNA virus sequences showing the location of each respective 2A-like (yellow rectangles). The nucleotide positions and size of each predicted polypeptide are represented by the numbers below and above the bars, respectively. The annotations of each viral sequence were included according to the NCBI. The nucleotide and protein accession numbers are presented forward and above each scheme, respectively. Representations of each genome are not in scale. This figure is presented in four parts.

Here, we registered 62 new 2A-like notifications in pssRNA viruses, as presented in Table 2 (underlined). The positions in each respective genome are shown in Figure 1.

In most pssRNA viruses, 2A/2A-like segments are used in primary polypeptide processing. The pssRNA viruses commonly possess one 2A/2A-like sequence, but some viruses have two, three, or even four motifs (Table 2). Many of them are members of the order Picornavirales, such as Picornaviridae, Dicistroviridae, and Iflaviridae. Currently, the Picornaviridae family has 63 assigned genera [28], but 2A/2A-like sequences have been found in viruses assigned or tentatively assigned to 15 genera: Aphthovirus, Avihepatovirus, Cardiovirus, Cosavirus, Crohivirus, Erbovirus, Hunninvirus, Limnipivirus, Mischivirus, Mosavirus, Parechovirus, Pasivirus, Senecavirus, Teschovirus, and Torchivirus.

In aphthoviruses and cardioviruses, the 2A-like region self-cleaves at its own C-terminus, meaning that the 2A-like polypeptide remains as a C-terminal extension of the upstream polyprotein (P1) until it is removed by secondary proteinase cleavage [8,9]. However, in parechoviruses, the 2A-like region has no protease or protease-like activity, and its apparent function is to alter host cell metabolism because it possesses a high homology to cellular protein H-rev107 that regulates cell proliferation (H-box 2A) [29].

In insect Iflaviruses, the 2A-like sequence separates the capsid and replicative protein domains. The Dicistroviridae family is composed of the Aparavirus, Cripavirus, and Triatovirus genera, in which the 2A-like sequences occur at the N-terminal region of the replicative protein open reading frame (ORF) [2,14].

Members of the Permutotetraviridae and Carmotetraviridae families (previously Tetraviridae), *Thosea asigna* virus and *Euprosterna elaeasa* virus, encode a 2A-like sequence at the N-terminus of the structural ORF [1]. The *Providence* virus has three 2A-like sequences, 2A_2_ and 2A_3_, located in the capsid protein precursor (VCAP), and 2A_1_ at the N-terminus of the p130 ORF, which encodes the viral replicase [30].

### 3.3. dsRNA Viruses

**Figure 2:**
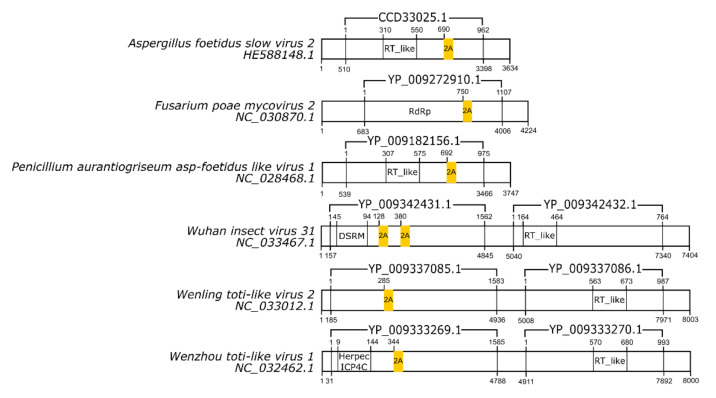
Schematic representation of double-stranded RNA virus sequences. Schematic representations of dsRNA virus sequences showing the location of each respective 2A-like (yellow rectangles). The nucleotide positions and size of each predicted polypeptide are represented by the numbers below and above the bars, respectively. The annotations of each viral sequence were made according to the information available at the NCBI. The nucleotide and protein accession numbers are located forward and above each scheme, respectively. Representations of each genome are not in scale.

Among the dsRNA viruses, 2A-like sequences not yet reported were found in six species. The new 2A-like sequences are underlined in Table 3, and their localization inside the genome is schematized in Figure 2.

In double-stranded viruses, 2A-like sequences are present in two families: Totiviridae and Reoviridae. In Totiviridae, 2A-like sequences are distributed in all representatives of the IMNV-like group [31]. These viruses predominantly infect arthropods, such as penaeid shrimp [32], mosquitoes [33,34], and the fruit fly *Drosophila melanogaster* [35], except for the golden shiner *Totivirus* that infects the fish *Notemigonus crysoleucas* [36]. The genome of IMNV-like viruses is composed of two ORFs, and the 2A-like sequences separate an RNA-binding protein of other putative proteins in ORF1 [37].

In the *Reoviridae* family, 2A-like sequences are found in cypoviruses and rotaviruses with 2A-like sequences in one of the segments encoding a non-structural protein. In *Operophtera brumata cypovirus 18* and *Bombyx mori cypovirus 1*, 2A-like sequences occur within segment 5. In type C rotaviruses, 2A-like sequences link the ssRNA-binding protein NSP3 to dsRNA-binding protein (dsRBP). In porcine and human rotavirus C, the 2A-like sequences are present at segment 6, although in the adult diarrhea virus, the sequence appears in segment 5 [1,2]. All cypoviruses and rotaviruses possess only one 2A-like sequence (Table 3).

### 3.4. nssRNA Virus

**Figure 3:**
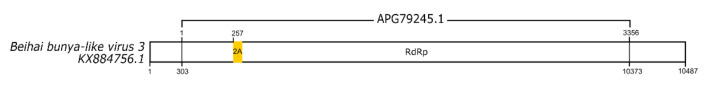
Schematic representation of a negative-sense single-strand RNA virus sequence. Schematic representations of nssRNA virus sequence showing the location of its respective 2A-like sequence (yellow rectangle). The nucleotide positions and size of the predicted polypeptide are represented by the numbers below and above the bars, respectively. The annotations of the viral sequence were made according to NCBI. The nucleotide and protein accession numbers are located forward and above the scheme, respectively. Representation of the genome are not to scale.

Surprisingly, one 2A-like motif (GDIEQNPGP) was found in a tentatively assigned virus of the Bunyaviridae family (Accession number: APG79245.1). This motif is located in the RNA-dependent RNA polymerase (RdRp) sequence (Figure 3). This is the first report of a 2A-like sequence in a nssRNA virus.

### 3.5. 2A/2A-Likes Sequences and Viral Evolution

Previous studies concerning RNA viruses and 2A-like peptides have reported that these sequences emerged independently during the evolution of viral families [2,14]. However, in a previous study [31], we showed sequences very similar to functional 2A-like sequences in some RNA viruses that could be the precursors of 2A sequences.

In particular, RNA viruses depend on the activity of RNA-dependent RNA polymerases. These enzymes have a significant error rate (10^−3^ to 10^−5^ mutations per inserted nucleotide) because they do not have exonucleotide review activity [38]. This results in a high degree of genetic heterogeneity in populations of RNA viruses, which are believed to favor adaptability to different environments and hosts [39]. Considering this, the 2A/2A-like sequences could have emerged by subsequent mutation events that ended in a cleavage function, providing the advantage of releasing more than one protein from the same ORF. Therefore, this could directly impact viral adaptation potential and viral infection mechanisms to favor their fitness in complex multicellular systems [31].

Yang et al. also suggested that picornaviruses with more complex infection mechanisms than other viruses of the same family have more than one 2A-like sequence in their genomes [14]. Taking this evidence into account, it seems that 2A/2A-like sequences may be a key element in viral genome evolution and, once acquired, its loss of function may impact virus effectivity.

### 3.6. Biotechnology Applications

Various approaches have been employed to co-express multiple proteins in cells, including the use of internal ribosomal entry site (IRES) elements [40,41], dual promoter systems [42,43], and transfection of multiple vectors [44]. Each of these is associated with several limitations, such as uneven or unreliable protein expression levels, silencing of some promoters [45,46], and increased toxicity to cells (with multiple transfections) [47].

Co-expression systems, including 2A/2A-like peptides, could be an alternative strategy for expressing multiple genes under the control of a single promoter. These constructs could have the additional advantage of producing proteins at near-stoichiometric levels, unlike IRES-mediated polycistronic expression, where ribosomes are independently recruited at distinct regions with the mRNA [1,4,48,49]. This necessitates the optimization of the system by testing several combinations of promoters and/or IRES and the order of genes within the expression cassette [46]. Furthermore, IRES activity can be affected by cell type, and variable expression can be observed in the downstream coding sequence [50].

2A/2A-like sequences have been used in a range of heterologous expression systems because of their cleavage capacity. These systems include viruses [51], yeasts [52,53], fungi [54,55,56], insect cells [57,58], plants [59], human HTK-143 cells [9], rabbit reticulocytes [60], HeLa cells [61], CHO cells [62], HEK293 cells [63], algae [64], and other animals [65,66,67].

In yeasts, more than two 2A sequences have been used to co-express proteins from the same vector. As seen in [68] and [69], three proteins were produced using this strategy in *S. cerevisiae*. Surprisingly, up to nine proteins have been linked and successfully co-translated and separated with 2A sequences in the yeast *Pichia pastoris* [70].

Researchers have also attempted to use 2A for multi-gene transformation in staple crops [71,72]. They can also be used for gene fusion, as seen in tomatoes, potatoes, and others [73,74].

To construct the co-expression vectors, the 2A/2A-like sequences are usually incorporated into an adenovirus [75], adeno-associated virus (AAV) [12], retrovirus [76], lentivirus [77,78], or plasmid vector [79,80]. Many other biotechnological applications that depend on the co-expression of multiple genes use 2A/2A-like sequences, e.g., the production of antibodies and antigens that can be used in vaccine production [80,81,82,83,84,85], observation of chromatin dynamics and genome (DNA and RNA) editing in the application of cell/gene therapies [78,79,86,87,88,89,90], and development of optogenetic tools [91,92,93]. More examples of viral 2As applications can be found in [94].

## 4. Conclusions

In this article, we reviewed the 2A/2A-like sequence distribution of viruses and described the occurrence of these motifs in viral species where these sequences have not been previously reported. These findings need to be confirmed through in vitro tests to verify they are active 2A-like sequences.

Because of its cleavage function, the 2A/2A-like sequences appear to directly affect the complexity of the viral genome, which plays a decisive role in viral evolution. Additionally, they are excellent alternatives for developing new biotechnological tools that depend on the expression of multiple products, such as vaccines, transgenic approaches, cell/gene therapy, and optogenetic tools.

## Figures and Tables

**Table 1:** Principal 2A/2A-like motifs described in literature and their cleavage efficiency.

Virus	Family	Motif	Cleavage Efficiency	References
*Euprosterna elaeasa virus* (EeV)	Alphatetraviridae	GDVEENPGP	~99%	[2,18]
*Providence virus* (PrV)	Alphatetraviridae	GDVESNPGP	~99%	[2]
*Providence virus* (PrV)	Alphatetraviridae	GDIEKNPGP	~94%	[2]
*Providence virus* (PrV)	Alphatetraviridae	GDVEKNPGP	~99%	[2]
*Thosea asigna virus* (TaV)	Alphatetraviridae	GDVEENPGP	~99%	[1]
*Acute bee paralysis virus* (ABPV)	Dicistroviridae	GDVETNPGP	~94%	[1,2]
*Cricket paralysis virus* (CrPV)	Dicistroviridae	GDVESNPGP	~90%	[1,2]
*Drosophila C virus* (DCV)	Dicistroviridae	GDVETNPGP	~95%	[1]
*Ectropis oblique picorna-like virus* (EoPV)	Iflaviridae	GDVESNPGP	~99%	[2,19]
*Ectropis oblique picorna-like virus* (EoPV)	Iflaviridae	GDIESNPGP	~99%	[2,19]
*Infectious flacherie virus* (IFV)	Iflaviridae	AGIESNPGP	~99%	[1,2]
*Perina nuda picorna-like virus* (PnPV)	Iflaviridae	GDVESNPGP	~99%	[2,20]
*Perina nuda picorna-like virus* (PnPV)	Iflaviridae	GDIESNPGP	~99%	[2,20]
*Encephalomyocarditis virus* (EMCV)	Picornaviridae	HDIETNPGP	~91%	[1,8]
*Equine rhinitis A virus* (ERAV)	Picornaviridae	GDVESNPGP	~99%	[1,21]
*Equine rhinitis B virus* (ERBV-1)	Picornaviridae	GDVELNPGP	~99%	[2,22]
*Foot-and-mouth disease virus* (FMDV)	Picornaviridae	GDVESNPGP	~99%	[8,10]
*Ljungan virus* (LV)	Picornaviridae	GDVETNPGP	~99%	[2,23]
*Porcine teschovirus 1* (PTV-1)	Picornaviridae	GDVEENPGP	~94%	[1,24]
*Saffold virus* (SAF-V)	Picornaviridae	HDVETNPGP	~99%	[2,25]
*Theiler’s murine encephalomyelitis virus* (TMEV)	Picornaviridae	HDVEMNPGP	~99%	[10]
*Bombyx mori reoviridae 1* (BmCPV-1)	Reoviridae	GDIESNPGP	~99%	[2,26]
*Human reoviridae C* (HurV-C)	Reoviridae	GDIELNPGP	~82%	[2]
*New adult diarrhea virus* (ADRV-N)	Reoviridae	ECIESNPGP	~97%	[2,27]
*Operophtera brumata reoviridae 18* (OpbuCPV-18)	Reoviridae	GDVESNPGP	~99%	[2]
*Porcine reoviridae A* (Porv-C)	Reoviridae	GDVELNPGP	~89%	[1,2]
*Infectious myonecrosis virus* (IMNV)	Unassigned Totiviridae	GDVESNPGP	~99%	[2,11]
*Infectious myonecrosis virus* (IMNV)	Unassigned Totiviridae	GDVEENPGP	~99%	[2,11]

**Table 2:** Positive-sense single-stranded RNA virus containing 2A-like motifs.

Accession Number	Virus	2A Motif	Taxon
YP_003620399.1	*Providence virus—*2A_1_	GDVEKNPGP	Carmotetraviridae
	*Providence virus—*2A_2_	GDVESNPGP	
	*Providence virus—*2A_3_	GDIEKNPGP	
NP_066241.1	*Acute bee paralysis virus*	GDVETNPGP	Dicistroviridae
YP_009252204.1	*Anopheles C virus*	GDVELNPGP	Dicistroviridae
NP_647481.1	*Cricket paralysis virus*	GDVESNPGP	Dicistroviridae
NP_044945.1	*Drosophila C virus*	GDVETNPGP	Dicistroviridae
AMO03208.1	*Empeyrat virus*	GDVELNPGP	Dicistroviridae
YP_008888535.1	*Formica exsecta virus 1*	GDIESNPGP	Dicistroviridae
YP_009221981.1	*Goose dicistrovirus*	GDVELNPGP	Dicistroviridae
ASS83246.1	*Israeli acute paralysis virus*	GDVEENPGP	Dicistroviridae
NP_851403.1	*Kashmir bee virus*	GDIELNPGP	Dicistroviridae
YP_009011065.1	*Fusarium graminearum hypovirus 1*	HDVEKNPGP	Hypoviridae
YP_009361829.1	*Diamond back moth iflavirus—*2A_1_	GDVESNPGP	Iflaviridae
	*Diamond back moth iflavirus—*2A_2_	GDVESNPGP	
NP_919029.1	*Ectropis obliqua picorna-like virus—*2A_1_	GDVESNPGP	Iflaviridae
	*Ectropis obliqua picorna-like virus—*2A_2_	GDIESNPGP	
NP_277061.1	*Perina nuda virus—*2A_1_	GDVESNPGP	Iflaviridae
	*Perina nuda virus—*2A_2_	GDIESNPGP	
YP_009010984.1	*Spodoptera exigua iflavirus 2*	GDVESNPGP	Iflaviridae
NP_573542.1	*Euprosterna elaeasa virus*	GDVEENPGP	Permutotetraviridae
AAC97195.1	*Thosea asigna virus*	GDVEENPGP	Permutotetraviridae
AXF38648.1	*Avihepatovirus sp.—*2A_1_	GDVESNPGP	Picornaviridae
	*Avihepatovirus sp.—*2A_2_	GDVESNPGP	
	*Avihepatovirus sp.—*2A_3_	GDVEPNPGP	
	*Avihepatovirus sp.—*2A_4_	GDVESNPGP	
AUX16868.1	*Avisivirus AVE052/AsV*	GDIEENPGP	Picornaviridae
YP_009345900.1	*Bat crohivirus*	GDIESNPGP	Picornaviridae
YP_006607894.1	*Bluegill picornavirus—*2A_1_	GDVESNPGP	Picornaviridae
	*Bluegill picornavirus—*2A_2_	GDVEQNPGP	
YP_006792625.1	*Bovine hungarovirus 1*	GDVELNPGP	Picornaviridae
YP_009116874.1	*Bovine picornavirus*	GDIESNPGP	Picornaviridae
AQX17368.1	*Bovine rhinitis B virus*	GDIESNPGP	Picornaviridae
ANN02879.1	*Bovine rhinitis B virus*	GDIETNPGP	Picornaviridae
YP_009352243.1	*Bovine rhinovirus 1*	GDVETNPGP	Picornaviridae
QEQ92497.1	*Burpengary virus*	GDVEQNPGP	Picornaviridae
ACG61138.2	*Cardiovirus D*	HDIETNPGP	Picornaviridae
AEJ86360.1	*Cardiovirus Hu/SIDS-347/DEU/2010*	HDIETNPGP	Picornaviridae
YP_008992026.1	*Carp picornavirus 1—*2A_1_	GDVEQNPGP	Picornaviridae
	*Carp picornavirus 1—*2A_2_	GDVESNPGP	
QMI57967.1	*Chestnut teal aalivirus*	GDVEENPGP	Picornaviridae
YP_002956074.1	*Cosavirus A*	GDIESNPGP	Picornaviridae
YP_002956076.1	*Cosavirus D*	GDIETNPGP	Picornaviridae
YP_009361830.1	*Cosavirus F*	GDVEENPGP	Picornaviridae
YP_009104360.1	*Crohivirus*	GDIESNPGP	Picornaviridae
YP_009345900.1	*Crohivirus B*	GDIESNPGP	Picornaviridae
YP_009026377.1	*Duck picornavirus GL/12—*2A_1_	GDVESNPGP	Picornaviridae
	*Duck picornavirus GL/12—*2A_2_	GDVEENPGP	
	*Duck picornavirus GL/12—*2A_3_	GDVEMNPGP	
	*Duck picornavirus GL/12—*2A_4_	GDIEQNPGP	
AAA43035.1	*Encephalomyocarditis virus*	HDIETNPGP	Picornaviridae
AKE44318.1	*Encephalomyocarditis virus*	HDVETNPGP	Picornaviridae
AGU38152.1	*Encephalomyocarditis virus*	HDVELNPGP	Picornaviridae
AFO66759.1	*Encephalomyocarditis virus type 2*	HDVETNPGP	Picornaviridae
NP_653077.1	*Equine rhinitis B virus 1*	GDVELNPGP	Picornaviridae
ANJ20934.1	*Equine rhinitis B virus 2*	GDVESNPGP	Picornaviridae
ANJ20932.1	*Erbovirus A*	GDVESNPGP	Picornaviridae
ANJ20933.1	*Erbovirus A*	GDVELNPGP	Picornaviridae
YP_009423853.1	*Falcon picornavirus—*2A_1_	GDVEENPGP	Picornaviridae
	*Falcon picornavirus—*2A_2_	GDVELNPGP	
AHL26986.1	*Fathead minnow picornavirus—*2A_1_	GDVEQNPGP	Picornaviridae
	*Fathead minnow picornavirus—*2A_2_	GDVESNPGP	
AYJ71467.2	*Feline hunnivirus*	GDVELNPGP	Picornaviridae
AAT01719.1	*Foot-and-mouth disease virus—*type A	GDVESNPGP	Picornaviridae
AFM56034.1	*Foot-and-mouth disease virus—*type O	GDVESNPGP	Picornaviridae
AAT01787.1	*Foot-and-mouth disease virus—*type SAT 1	GDVESNPGP	Picornaviridae
AFE84748.1	*Foot-and-mouth disease virus—*type SAT 2	GDVESNPGP	Picornaviridae
AAT01795.1	*Foot-and-mouth disease virus—*type SAT 3	GDVESNPGP	Picornaviridae
AIB06813.1	*Genet fecal theilovirus*	HDVEMNPGP	Picornaviridae
YP_009026376.1	*Human cosavirus*	GDIETNPGP	Picornaviridae
AFJ04537.1	*Human cosavirus A20*	GDIESNPGP	Picornaviridae
YP_002956075.1	*Human cosavirus B*	HDIETNPGP	Picornaviridae
ADF28539.1	*Human TMEV-like cardiovirus*	HDIETNPGP	Picornaviridae
AMT85188.1	*Hunnivirus*	GDVEENPGP	Picornaviridae
YP_009118270.1	*Lesavirus 2*	GDIEPNPGP	Picornaviridae
ACJ48052.1	*Ljungan virus*	GDVEENPGP	Picornaviridae
AVX29482.1	*Marmot mosavirus—*2A_1_	GDVETNPGP	Picornaviridae
	*Marmot mosavirus—*2A_2_	GDVETNPGP	
ANX14418.1	*Mengo virus*	HDVETNPGP	Picornaviridae
YP_009361319.1	*Miniopterus schreibersii picornavirus 1*	GDVEENPGP	Picornaviridae
AWC68493.1	*Mischivirus B*	GDIEENPGP	Picornaviridae
YP_009026384.1	*Mosavirus A2*	GDVESNPGP	Picornaviridae
YP_009109563.1	*Norway rat hunnivirus*	GDVELNPGP	Picornaviridae
ADO85550.2	*Ovine hungarovirus*	GDVELNPGP	Picornaviridae
AIU94297.1	*Pasivirus A*	GDVEQNPGP	Picornaviridae
SNQ28005.1	*Pasivirus A*	GDIEQNPGP	Picornaviridae
APA29021.1	*Picornaviridae sp. rodent*	GDVELNPGP	Picornaviridae
ADN52625.1	*Porcine encephalomyocarditis virus*	HDIETNPGP	Picornaviridae
AAK12398.1	*Porcine teschovirus 1*	GDVEENPGP	Picornaviridae
AAK12413.1	*Porcine teschovirus 10*	GDVEENPGP	Picornaviridae
AAK12390.1	*Porcine teschovirus 11*	GDVEENPGP	Picornaviridae
AAK12381.1	*Porcine teschovirus 2*	GDVEENPGP	Picornaviridae
AAK12382.1	*Porcine teschovirus 3*	GDVEENPGP	Picornaviridae
AGB67759.1	*Porcine teschovirus 4*	GDVEENPGP	Picornaviridae
ACT66681.1	*Porcine teschovirus 5*	GDVEENPGP	Picornaviridae
AAK12409.1	*Porcine teschovirus 6*	GDVEENPGP	Picornaviridae
AAK12386.1	*Porcine teschovirus 7*	GDVEENPGP	Picornaviridae
AAK12388.1	*Porcine teschovirus 9*	GDVEENPGP	Picornaviridae
QHX40840.1	*Porcine teschovirus 22*	GDIEENPGP	Picornaviridae
ACD67870.1	*Rat theilovirus 1*	HDVETNPGP	Picornaviridae
AWK02689.1	*Rattus tanezumi hunnivirus*	GDVEENPGP	Picornaviridae
AWK02688.1	*Rattus tanezumi parechovirus—*2A_1_	GDVEENPGP	Picornaviridae
	*Rattus tanezumi parechovirus—*2A_2_	GDVEENPGP	
ACO92353.1	*Saffold virus*	HDIETNPGP	Picornaviridae
YP_001210296.2	*Saffold virus*	HDVETNPGP	Picornaviridae
APZ85840.1	*Senecavirus A*	GDIETNPGP	Picornaviridae
AHW57724.1	*Sikhote-Alin virus*	HDVEMNPGP	Picornaviridae
AUK47911.1	*Swine pasivirus SPaV1/US/17-50816IA60467-1/2001*	GDVEQNPGP	Picornaviridae
BAU71153.1	*Swine picornavirus*	GDVEENPGP	Picornaviridae
NP_653143.1	*Teschovirus A*	GDVEENPGP	Picornaviridae
ACG55799.1	*Theiler’s encephalomyelitis virus*	HDVETNPGP	Picornaviridae
BAC58035.1	*Theiler’s-like virus of rats*	HDVETNPGP	Picornaviridae
AIY68187.1	*Tortoise picornavirus*	GDVEVNPGP	Picornaviridae
AIY68186.1	*Tortoise picornavirus*	GDVEQNPGP	Picornaviridae
ACG55801.1	*Vilyuisk human encephalomyelitis virus*	HDVEMNPGP	Picornaviridae
AVM87411.1	*Yili teratoscincus roborowskii picornavirus 2*	GDVEQNPGP	Picornaviridae
YP_009329817.1	*Bivalve RNA virus G1*	GDVETNPGP	Unassigned Dicistroviridae
QNL09596.1	*Clinch dicistro-like virus 2—2A_1_*	GDVEMNPGP	Unassigned Dicistroviridae
	*Clinch dicistro-like virus 2—2A_2_*	GDVETNPGP	
QJI52079.1	*Dicistroviridae sp.*	GDVEMNPGP	Unassigned Dicistroviridae
AYQ66681.1	*Drosophila kikkawai virus 1*	GDVELNPGP	Unassigned Dicistroviridae
YP_009336571.1	*Hubei diptera virus 1*	GDVELNPGP	Unassigned Dicistroviridae
YP_009336583.1	*Hubei picorna-like virus 16*	GDVELNPGP	Unassigned Dicistroviridae
YP_009336853.1	*Hubei picorna-like virus 17*	GDVELNPGP	Unassigned Dicistroviridae
QKF95572.1	*Leibnitzia anandria dicistrovirus*	GDIEENPGP	Unassigned Dicistroviridae
AXA52579.1	*Linepithema humile virus 1*	GDIELNPGP	Unassigned Dicistroviridae
QIU80542.1	*Phenacoccus solenopsis virus*	GDIEENPGP	Unassigned Dicistroviridae
YP_009336743.1	*Wenling crustacean virus 3*	GDVEENPGP	Unassigned Dicistroviridae
YP_009333180.1	*Wenling picorna-like virus 2*	GDIELNPGP	Unassigned Dicistroviridae
YP_009342327.1	*Wuhan insect virus 11*	GDIEANPGP	Unassigned Dicistroviridae
YP_009329857.1	*Beihai hepe-like virus 4*	GDIESNPGP	Unassigned Hepeviridae
QDY81493.1	*Bipolaris oryzae hypovirus 1*	GDVEANPGP	Unassigned Hypoviridae
YP_009337372.1	*Hubei picorna-like virus 43*	GDIESNPGP	Unassigned Iflaviridae
QKN89050.1	*Iflaviridae sp.—*2A_1_	GDVESNPGP	Unassigned Iflaviridae
	*Iflaviridae sp.—*2A_2_	GDIESNPGP	
AWK77896.1	*Perth bee virus 3*	GDVETNPGP	Unassigned Iflaviridae
YP_009336821.1	*Wenzhou picorna-like virus 49*	HDVELNPGP	Unassigned Iflaviridae
AVM87450.1	*Guangdong spotted longbarbel catfish picornavirus—2A_1_*	GDVEENPGP	Unassigned Picornavirales
	*Guangdong spotted longbarbel catfish picornavirus—2A_2_*	GDIESNPGP	
	*Guangdong spotted longbarbel catfish picornavirus—2A_3_*	GDVERNPGP	
ASG92543.1	*Picornavirales Q_sR_OV_036*	GDVEANPGP	Unassigned Picornavirales
ASG92538.1	*Picornavirales Q_sR_OV_042*	GDIEENPGP	Unassigned Picornavirales
ATY47693.1	*Picornavirales sp.*	GDVEENPGP	Unassigned Picornavirales
ATY47707.1	*Picornavirales sp.*	GDVELNPGP	Unassigned Picornavirales
AWK02666.1	*Rhinolophus sinicus picornavirus*	GDIEENPGP	Unassigned Picornavirales
QQP18688.1	*Soybean thrips picorna-like virus 7*	GDVETNPGP	Unassigned Picornavirales
AWK02669.1	*Suncus murinus picornavirus*	GDVETNPGP	Unassigned Picornavirales
AWK77886.1	*Victoria bee virus 1*	GDVETNPGP	Unassigned Picornavirales
AWK77887.1	*Victoria bee virus 2*	GDIETNPGP	Unassigned Picornavirales
AVM87443.1	*Wenling thamnaconus septentrionalis picornavirus*	GDIESNPGP	Unassigned Picornavirales
AVM87419.1	*Western African lungfish picornavirus*	GDVEENPGP	Unassigned Picornavirales
AVM87438.1	*Wuhan carp picornavirus—*2A_1_	GDVESNPGP	Unassigned Picornavirales
	*Wuhan carp picornavirus—*2A_2_	GDVESNPGP	
	*Wuhan carp picornavirus—*2A_3_	GDVESNPGP	
ANN02882.1	*Bovine rhinitis B virus 5*	GDVETNPGP	Unassigned Picornaviridae
AQM40272.1	*Human cosavirus (Cosavirus-zj-1)*	GDVEENPGP	Unassigned Picornaviridae
AWG94399.1	*Human cosavirus E/D*	GDVEENPGP	Unassigned Picornaviridae
AVX29481.1	*Marmot cardiovirus*	HDVETNPGP	Unassigned Picornaviridae
AWK02672.1	*Niviventer confucianus hunnivirus*	GDVELNPGP	Unassigned Picornaviridae
AFV31450.1	*Parechovirus-like virus*	GDVEQNPGP	Unassigned Picornaviridae
QBH68005.1	*Parechovirus sp. QAPp32*	GDVEENPGP	Unassigned Picornaviridae
QKE55061.1	*Picornaviridae sp.*	GDIEENPGP	Unassigned Picornaviridae
QKE55028.1	*Picornaviridae sp.—*2A_1_	GDVESNPGP	Unassigned Picornaviridae
	*Picornaviridae sp.—*2A_2_	GDVEQNPGP	
	*Picornaviridae sp.—*2A_3_	GDVESNPGP	
QIM74091.1	*Picornaviridae sp.*	HDVETNPGP	Unassigned Picornaviridae
YP_009336671.1	*Wenzhou picorna-like virus 48—2A_1_*	GDIEENPGP	Unassigned Picornaviridae
	*Wenzhou picorna-like virus 48—2A_2_*	GDIESNPGP	
	*Wenzhou picorna-like virus 48—2A_3_*	GDIEENPGP	
AZT88626.1	*Aspergillus homomorphus yadokarivirus 1*	GDIEENPGP	Unassigned pssRNA
APG77930.1	*Beihai picorna-like virus 76*	GDVETNPGP	Unassigned pssRNA
YP_009333551.1	*Beihai picorna-like virus 85*	GDVETNPGP	Unassigned pssRNA
AYN75548.1	*Halhan virus 1*	GDVEQNPGP	Unassigned pssRNA
AZT88627.1	*Penicillium digitatum yadokarivirus 1*	GDVETNPGP	Unassigned pssRNA
QOI17269.1	*Picoa juniperi yado-kari virus 1*	GDIESNPGP	Unassigned pssRNA
QHD64758.1	*Plasmopara viticola lesion associated yadokari virus 1*	GDIEENPGP	Unassigned pssRNA
QIJ25855.1	*Warroolaba Creek virus 2*	GDVETNPGP	Unassigned pssRNA
AVD68673.2	*Yado-kari virus 2*	GDVEENPGP	Unassigned pssRNA

Underlined names correspond to sequences that had no 2A sequence described before this study.

**Table 3:** Double-stranded RNA viruses identified in this study containing 2A-like motifs.

Accession Number	Virus	2A Motif	Taxon
AAU88188.1	*Adult diarrhea virus*	ECIESNPGP	Reoviridae
BAB20437.1	*Bombyx mori cypovirus 1*	GDIESNPGP	Reoviridae
BAO73973.1	*Bovine rotavirus C*	GDVELNPGP	Reoviridae
AAO32344.1	*Dendrolimus punctatus cypovirus 1*	GDVESNPGP	Reoviridae
BAU80889.1	*Human rotavirus C*	GDIELNPGP	Reoviridae
AAK73524.1	*Lymantria dispar cypovirus 1*	GDVESNPGP	Reoviridae
ABB17215.1	*Operophtera brumata cypovirus 18*	GDVESNPGP	Reoviridae
BAV31546.1	*Porcine rotavirus C*	GDVELNPGP	Reoviridae
QBJ02264.1	*Porcine rotavirus H*	GDVELNPGP	Reoviridae
AQX34666.1	*Rotavirus I*	GDIESNPGP	Reoviridae
CCD33025.1	*Aspergillus foetidus slow virus 2*	GDIEENPGP	Unassigned dsRNA
YP_009272910.1	*Fusarium poae mycovirus 2*	GDIEENPGP	Unassigned dsRNA
YP_009182156.1	*Penicillium aurantiogriseum asp-foetidus like virus 1*	GDIEENPGP	Unassigned dsRNA
YP_009342431.1	*Wuhan insect virus 31—2A_1_*	GDVELNPGP	Unassigned dsRNA
	*Wuhan insect virus 31—2A_2_*	GDVERNPGP	
YP_003934933.1	*Armigeres subalbatus*	GDVESNPGP	Unassigned Totiviridae
YP_009256208.1	*Golden shiner totivirus*	GDIESNPGP	Unassigned Totiviridae
AIC34742.2	*Penaeid shrimp infectious myonecrosis virus—*2A_1_	GDVESNPGP	Unassigned Totiviridae
	*Penaeid shrimp infectious myonecrosis virus—*2A_2_	GDVEENPGP	
YP_009337085.1	*Wenling toti-like virus 2*	GDIETNPGP	Unassigned Totiviridae
YP_009333269.1	*Wenzhou toti-like virus 1*	GDVEMNPGP	Unassigned Totiviridae

Underlined names correspond to new findings.

## References

[1] Donnelly M.L.L., Hughes L.E., Luke G., Mendoza H., Ten Dam E., Gani D., Ryan M.D. (2001). The “cleavage” activities of foot-and-mouth disease virus 2A site-directed mutants and naturally occurring “2A-like” sequences. J. Gen. Virol..

[2] Luke G.A., de Felipe P., Lukashev A., Kallioinen S.E., Bruno E.A., Ryan M.D. (2008). Occurrence, function and evolutionary origins of ‘2A-like’ sequences in virus genomes. J. Gen. Virol..

[3] Ryan M.D., Donnelly M., Lewis A., Mehrotra A.P., Wilkie J., Gani D. (1999). A model for nonstoichiometric, cotranslational protein scission in eukaryotic ribosomes. Bioorg. Chem..

[4] Donnelly M.L.L., Luke G., Mehrotra A., Li X., Hughes L.E., Gani D., Ryan M.D. (2001). Analysis of the aphthovirus 2A/2B polyprotein “cleavage” mechanism indicates not a proteolytic reaction, but a novel translational effect: A putative ribosomal “skip”. J. Gen. Virol..

[5] Atkins J.F., Wills N.M., Loughran G., Wu C.Y., Parsawar K., Ryan M.D., Wang C.H., Nelson C.C. (2007). A case for “StopGo”: Reprogramming translation to augment codon meaning of GGN by promoting unconventional termination (Stop) after addition of glycine and then allowing continued translation (Go). RNA.

[6] Brown J.D., Ryan M.D., Atkins J.F., Gesteland R.F. (2010). Ribosome “Skipping”: “Stop-Carry On” or “StopGo” Translation. Recoding: Expansion of Decoding Rules Enriches Gene Expression.

[7] de Felipe P. (2004). Skipping the co-expression problem: The new 2A “CHYSEL” technology. Genet. Vaccines Ther..

[8] Ryan M.D., King A.M.Q., Thomas G.P. (1991). Cleavage of foot-and-mouth disease virus polyprotein is mediated by residues located within a 19 amino acid sequence. J. Gen. Virol..

[9] Ryan M.D., Drew J. (1994). Foot-and-mouth disease virus 2A oligopeptide mediated cleavage of an artificial polyprotein. EMBO J..

[10] Donnelly M.L.L., Gani D., Flint M., Monaghan S., Ryan M.D. (1997). The cleavage activities of aphthovirus and cardiovirus 2A proteins. J. Gen. Virol..

[11] Nibert M.L. (2007). “2A-like” and “shifty heptamer” motifs in *penaeid shrimp infectious myonecrosis virus*, a monosegmented double-stranded RNA virus. J. Gen. Virol..

[12] Lewis J.E., Brameld J.M., Hill P., Barrett P., Ebling F.J.P., Jethwa P.H. (2015). The use of a viral 2A sequence for the simultaneous over-expression of both the vgf gene and enhanced green fluorescent protein (eGFP) in vitro and in vivo. J. Neurosci. Methods.

[13] Doronina V.A., de Felipe P., Wu C., Sharma P., Sachs M.S., Ryan M.D., Brown J.D. (2008). Dissection of a co-translational nascent chain separation event. Biochem. Soc. Trans..

[14] Yang X., Cheng A., Wang M., Jia R., Sun K., Pan K., Yang Q., Wu Y., Zhu D., Chen S. (2017). Structures and corresponding functions of five types of picornaviral 2A proteins. Front. Microbiol..

[15] Luke G.A., Escuin H., De Felipe P., Ryan M. (2009). 2A to the Fore–Research, Technology and Applications 2A to the Fore–Research, Technology and Applications. Biotechnol. Genet. Eng. Rev..

[16] Luke G.A., Pathania U.S., Roulston C., De Felipe P., Ryan M.D. (2014). DxExNPGP-Motives for the motif. Recent Research Developments in Virology.

[17] Luke G.A., Ryan M.D. (2013). The protein coexpression problem in biotechnology and biomedicine: Virus 2A and 2A-like sequences provide a solution. Future Virol..

[18] Gorbalenya A.E., Pringle F.M., Zeddam J.L., Luke B.T., Cameron C.E., Kalmakoff J., Hanzlik T.N., Gordon K.H.J., Ward V.K. (2002). The palm subdomain-based active site is internally permuted in viral RNA-dependent RNA polymerases of an ancient lineage. J. Mol. Biol..

[19] Wang X., Zhang J., Lu J., Yi F., Liu C., Hu Y. (2004). Sequence analysis and genomic organization of a new insect picorna-like virus, Ectropis obliqua picorna-like virus, isolated from Ectropis obliqua. J. Gen. Virol..

[20] Wu C.Y., Lo C.F., Huang C.J., Yu H.T., Wang C.H. (2002). The complete genome sequence of Perina nuda picorna-like virus, an insect-infecting RNA virus with a genome organization similar to that of the mammalian picornaviruses. Virology.

[21] Li F., Browning G.F., Studdert M.J., Crabb B.S. (1996). Equine rhinovirus 1 is more closely related to foot-and-mouth disease virus than to other picornaviruses. Proc. Natl. Acad. Sci. USA.

[22] Wutz G., Auer H., Nowotny N., Grosse B., Skern T., Kuechler E. (1996). Equine rhinovirus serotypes 1 and 2: Relationship to each other and to aphthoviruses and cardioviruses. J. Gen. Virol..

[23] Lindberg A.M., Johansson S. (2002). Phylogenetic analysis of Ljungan virus and A-2 plaque virus, new members of the Picornaviridae. Virus Res..

[24] Doherty M., Todd D., McFerran N., Hoey E.M. (1999). Sequence analysis of a *porcine enterovirus serotype 1* isolate: Relationships with other picornaviruses. J. Gen. Virol..

[25] Jones M.S., Lukashov V.V., Ganac R.D., Schnurr D.P. (2007). Discovery of a novel human picornavirus in a stool sample from a pediatric patient presenting with fever of unknown origin. J. Clin. Microbiol..

[26] Hagiwara K., Kobayashi J., Tomita M., Yoshimura T. (2001). Nucleotide sequence of genome segment 5 from Bombyx mori cypovirus 1. Arch. Virol..

[27] Yang H., Makeyev E.V., Kang Z., Ji S., Bamford D.H., Van Dijk A.A. (2004). Cloning and sequence analysis of dsRNA segments 5, 6 and 7 of a novel non-group A, B, C adult rotavirus that caused an outbreak of gastroenteritis in China. Virus Res..

[28] Zell R., Delwart E., Gorbalenya A.E., Hovi T., King A.M.Q., Knowles N.J., Lindberg A.M., Pallansch M.A., Palmenberg A.C., Reuter G. (2017). ICTV virus taxonomy profile: *Picornaviridae*. J. Gen. Virol..

[29] Hughes P.J., Stanway G. (2000). The 2A proteins of three diverse picornaviruses are related to each other and to the H-rev107 family of proteins involved in the control of cell proliferation. J. Gen. Virol..

[30] Walter C.T., Pringle F.M., Nakayinga R., De Felipe P., Ryan M.D., Ball L.A., Dorrington R.A. (2010). Genome organization and translation products of *Providence virus*: Insight into a unique tetravirus. J. Gen. Virol..

[31] de Lima J.G.S., Teixeira D.G., Freitas T.T., Lima J.P.M.S., Lanza D.C.F. (2019). Evolutionary origin of 2A-like sequences in *Totiviridae* genomes. Virus Res..

[32] Poulos B.T., Tang K.F.J., Pantoja C.R., Bonami J.R., Lightner D.V. (2006). Purification and characterization of infectious myonecrosis virus of penaeid shrimp. J. Gen. Virol..

[33] Zhai Y., Attoui H., Mohd Jaafar F., Wang H.-Q., Cao Y.-X., Fan S.-P., Sun Y.-X., Liu L.-D., Mertens P.P.C., Meng W.-S. (2010). Isolation and full-length sequence analysis of *Armigeres subalbatus* totivirus, the first totivirus isolate from mosquitoes representing a proposed novel genus (*Artivirus*) of the family *Totiviridae*. J. Gen. Virol..

[34] Isawa H., Kuwata R., Hoshino K., Tsuda Y., Sakai K., Watanabe S., Nishimura M., Satho T., Kataoka M., Nagata N. (2011). Identification and molecular characterization of a new nonsegmented double-stranded RNA virus isolated from *Culex mosquitoes* in Japan. Virus Res..

[35] Wu Q., Luo Y., Lu R., Lau N., Lai E.C., Li W.X., Ding S.W. (2010). Virus discovery by deep sequencing and assembly of virus-derived small silencing RNAs. Proc. Natl. Acad. Sci. USA.

[36] Virol A., Mor S.K., Benjamin N., Phelps D. (2016). Molecular detection of a novel totivirus from golden shiner (*Notemigonus crysoleucas*) baitfish in the USA. Arch. Virol..

[37] Danielle M., Dantas A., Henrique G., Cavalcante O., Oliveira R.A.C.C., Lanza D.C.F.F., Dantas M.D.A., Cavalcante G.H.O., Oliveira R.A.C.C., Lanza D.C.F.F. (2016). New insights about ORF1 coding regions support the proposition of a new genus comprising arthropod viruses in the family *Totiviridae*. Virus Res..

[38] Modrow S., Falke D., Truyen U., Schätzl H. (2013). How Do Mutations Lead to the Emergence of Novel Viruses?.

[39] Orton R.J., Wright C.F., King D.P., Haydon D.T. (2019). Estimating viral bottleneck sizes for FMDV transmission within and between hosts and implications for the rate of viral evolution. Interface Focus.

[40] Khodamoradi S., Shenagari M., Kheiri M.T., Sabahi F., Jamali A., Heidari A., Ashrafkhani B. (2018). IRES-based co-expression of influenza virus conserved genes can promote synergistic antiviral effects both in vitro and in vivo. Arch. Virol..

[41] Akiyoshi S., Ishii T., Bai Z., Mombaerts P. (2018). Subpopulations of vomeronasal sensory neurons with coordinated coexpression of type 2 vomeronasal receptor genes are differentially dependent on Vmn2r1. Eur. J. Neurosci..

[42] Zhang K., Su L., Duan X., Liu L., Wu J. (2017). High-level extracellular protein production in *Bacillus subtilis* using an optimized dual-promoter expression system. Microb. Cell Fact..

[43] Bayat H., Hossienzadeh S., Pourmaleki E., Ahani R., Rahimpour A. (2018). Evaluation of different vector design strategies for the expression of recombinant monoclonal antibody in CHO cells. Prep. Biochem. Biotechnol..

[44] Takahashi K., Yamanaka S. (2006). Induction of Pluripotent Stem Cells from Mouse Embryonic and Adult Fibroblast Cultures by Defined Factors. Cell.

[45] Tang W., Ehrlich I., Wolff S.B.E., Michalski A.M., Wölfl S., Hasan M.T., Lüthi A., Sprengel R. (2009). Faithful expression of multiple proteins via 2A-peptide self-processing: A versatile and reliable method for manipulating brain circuits. J. Neurosci..

[46] Radcliffe P.A., Mitrophanous K.A. (2004). Multiple gene products from a single vector: “Self-cleaving” 2A peptides. Gene Ther..

[47] Arbab A.S., Yocum G.T., Wilson L.B., Parwana A., Jordan E.K., Kalish H., Frank J.A. (2004). Comparison of transfection agents in forming complexes with ferumoxides, cell labeling efficiency, and cellular viability. Mol. Imaging.

[48] de Felipe P., Luke G.A., Hughes L.E., Gani D., Halpin C., Ryan M.D. (2006). E unum pluribus: Multiple proteins from a self-processing polyprotein. Trends Biotechnol..

[49] Hellen C.U.T., Sarnow P. (2001). Internal ribosome entry sites in eukaryotic mRNA molecules. Genes Dev..

[50] Bouabe H., Fässler R., Heesemann J. (2008). Improvement of reporter activity by IRES-mediated polycistronic reporter system. Nucleic Acids Res..

[51] Momose F., Morikawa Y. (2016). Polycistronic expression of the influenza A virus RNA-dependent RNA polymerase by using the Thosea asigna virus 2A-like self-processing sequence. Front. Microbiol..

[52] Sun Y.F., Lin Y., Zhang J.H., Zheng S.P., Ye Y.R., Liang X.X., Han S.Y. (2012). Double *Candida antarctica* lipase B co-display on Pichia pastoris cell surface based on a self-processing foot-and-mouth disease virus 2A peptide. Appl. Microbiol. Biotechnol..

[53] Wang S., Yao Q., Tao J., Qiao Y., Zhang Z. (2007). Co-ordinate expression of glycine betaine synthesis genes linked by the FMDV 2A region in a single open reading frame in *Pichia pastoris*. Appl. Microbiol. Biotechnol..

[54] Subramanian V., Schuster L.A., Moore K.T., Ii L.E.T., Baker J.O., Wall T.A., Vander Linger J.G., Himmel M.E., Decker S.R. (2017). Biotechnology for Biofuels A versatile 2A peptide - based bicistronic protein expressing platform for the industrial cellulase producing fungus, Trichoderma reesei. Biotechnol. Biofuels.

[55] Schuetze T., Meyer V. (2017). Polycistronic gene expression in *Aspergillus niger*. Microb. Cell Fact..

[56] Li F., Liu Q., Li X., Zhang C., Li J., Sun W., Liu D., Xiao D., Tian C. (2020). Construction of a new thermophilic fungus *Myceliophthora thermophila* platform for enzyme production using a versatile 2A peptide strategy combined with efficient CRISPR-Cas9 system. Biotechnol. Lett..

[57] Osborn M.J., Panoskaltsis-Mortari A., McElmurry R.T., Bell S.K., Vignali D.A.A., Ryan M.D., Wilber A.C., McIvor R.S., Tolar J., Blazar B.R. (2005). A picornaviral 2A-like sequence-based tricistronic vector allowing for high-level therapeutic gene expression coupled to a dual-reporter system. Mol. Ther..

[58] Schwirz J., Yan Y., Franta Z., Schetelig M.F. (2020). Bicistronic expression and differential localization of proteins in insect cells and *Drosophila suzukii* using picornaviral 2A peptides. Insect Biochem. Mol. Biol..

[59] Zhang B., Rapolu M., Kumar S., Gupta M., Liang Z., Han Z., Williams P., Su W.W. (2017). Coordinated protein co-expression in plants by harnessing the synergy between an intein and a viral 2A peptide. Plant Biotechnol. J..

[60] de Felipe P., Hughes L.E., Ryan M.D., Brown J.D. (2003). Co-translational, intraribosomal cleavage of polypeptides by the foot-and-mouth disease virus 2A peptide. J. Biol. Chem..

[61] de Felipe P., Luke G.A., Brown J.D., Ryan M.D. (2010). Inhibition of 2A-mediated “cleavage” of certain artificial polyproteins bearing N-terminal signal sequences. Biotechnol. J..

[62] Li Y., Wang M., Wang T., Wei Y., Guo X., Mi C., Zhao C., Cao X., Dou Y. (2020). Effects of different 2A peptides on transgene expression mediated by tricistronic vectors in transfected CHO cells. Mol. Biol. Rep..

[63] Shin S., Kim S.H., Shin S.W., Grav L.M., Pedersen L.E., Lee J.S., Lee G.M. (2020). Comprehensive Analysis of Genomic Safe Harbors as Target Sites for Stable Expression of the Heterologous Gene in HEK293 Cells. ACS Synth. Biol..

[64] Rasala B.A., Lee P.A., Shen Z., Briggs S.P., Mendez M., Mayfield S.P. (2012). Robust expression and secretion of xylanase1 in *Chlamydomonas reinhardtii* by fusion to a selection gene and processing with the FMDV 2A peptide. PLoS ONE.

[65] Jeong I., Kim E., Seong J.Y., Park H.C. (2019). Overexpression of Spexin 1 in the Dorsal Habenula Reduces Anxiety in Zebrafish. Front. Neural Circuits.

[66] Pontes-Quero S., Fernández-Chacón M., Luo W., Lunella F.F., Casquero-Garcia V., Garcia-Gonzalez I., Hermoso A., Rocha S.F., Bansal M., Benedito R. (2019). High mitogenic stimulation arrests angiogenesis. Nat. Commun..

[67] Wang Y., Wang F., Xu S., Wang R., Chen W., Hou K., Tian C., Wang F., Zhao P., Xia Q. (2019). Optimization of a 2A self-cleaving peptide-based multigene expression system for efficient expression of upstream and downstream genes in silkworm. Mol. Genet. Genomics.

[68] Beekwilder J., Van Rossum H.M., Koopman F., Sonntag F., Buchhaupt M., Schrader J., Hall R.D., Bosch D., Pronk J.T., Van Maris A.J.A. (2014). Polycistronic expression of a β-carotene biosynthetic pathway in Saccharomyces cerevisiae coupled to β-ionone production. J. Biotechnol..

[69] Park M., Kang K., Park S., Kim Y.S., Ha S.H., Lee S.W., Ahn M.J., Bae J.M., Back K. (2008). Expression of serotonin derivative synthetic genes on a single self-processing polypeptide and the production of serotonin derivatives in microbes. Appl. Microbiol. Biotechnol..

[70] Geier M., Fauland P., Vogl T., Glieder A. (2015). Compact multi-enzyme pathways in *P. pastoris*. Chem. Commun..

[71] Park S., Kang K., Kim Y.S., Back K. (2009). Endosperm-specific expression of tyramine N-hydroxycinnamoyltransferase and tyrosine decarboxylase from a single self-processing polypeptide produces high levels of tyramine derivatives in rice seeds. Biotechnol. Lett..

[72] Quilis J., López-García B., Meynard D., Guiderdoni E., San Segundo B. (2014). Inducible expression of a fusion gene encoding two proteinase inhibitors leads to insect and pathogen resistance in transgenic rice. Plant Biotechnol. J..

[73] Yeo E.T., Kwon H.B., Han S.E., Lee J.T., Ryu J.C., Byun M.O. (2000). Genetic engineering of drought resistant potato plants by introduction of the trehalose-6-phosphate synthase (TPS1) gene from *Saccharomyces cerevisiae*. Mol. Cells.

[74] Ralley L., Enfissi E.M.A., Misawa N., Schuch W., Bramley P.M., Fraser P.D. (2004). Metabolic engineering of ketocarotenoid formation in higher plants. Plant J..

[75] Chu V.T., Weber T., Wefers B., Wurst W., Sander S., Rajewsky K., Kühn R. (2015). Increasing the efficiency of homology-directed repair for CRISPR-Cas9-induced precise gene editing in mammalian cells. Nat. Biotechnol..

[76] Browne E.P. (2015). An Interleukin-1 Beta-Encoding Retrovirus Exhibits Enhanced Replication In Vivo. J. Virol..

[77] Yi G., Choi J.G., Bharaj P., Abraham S., Dang Y., Kafri T., Alozie O., Manjunath M.N., Shankar P. (2014). CCR5 gene editing of resting CD4+ T cells by transient ZFN expression from HIV envelope pseudotyped nonintegrating lentivirus confers HIV-1 resistance in humanized mice. Mol. Ther.-Nucleic Acids.

[78] Peng Y., Yang T., Tang X., Chen F., Wang S. (2020). Construction of an Inducible CRISPR/Cas9 System for CXCR4 Gene and Demonstration of its Effects on MKN-45 Cells. Cell Biochem. Biophys..

[79] Fang Y., Stroukov W., Cathomen T., Mussolino C. (2020). Chimerization enables gene synthesis and lentiviral delivery of customizable tale-based effectors. Int. J. Mol. Sci..

[80] Mizote Y., Masumi-Koizumi K., Katsuda T., Yamaji H. (2020). Production of an antibody Fab fragment using 2A peptide in insect cells. J. Biosci. Bioeng..

[81] Arevalo-Villalobos J.I., Govea-Alonso D.O., Bañuelos-Hernández B., González-Ortega O., Zarazúa S., Rosales-Mendoza S. (2020). Inducible expression of antigens in plants: A study focused on peptides related to multiple sclerosis immunotherapy. J. Biotechnol..

[82] Chen T.H., Hu C.C., Liao J.T., Lee Y.L., Huang Y.W., Lin N.S., Lin Y.L., Hsu Y.H. (2017). Production of Japanese encephalitis virus antigens in plants using bamboo mosaic virus-based vector. Front. Microbiol..

[83] Lin Y., Hung C.Y., Bhattacharya C., Nichols S., Rahimuddin H., Kittur F.S., Leung T.C., Xie J. (2018). An effective way of producing fully assembled antibody in transgenic tobacco plants by linking heavy and light chains via a self-cleaving 2a peptide. Front. Plant Sci..

[84] Chen L., Yang X., Luo D., Yu W. (2016). Efficient Production of a Bioactive Bevacizumab monoclonal antibody using the 2A self-cleavage peptide in transgenic rice callus. Front. Plant Sci..

[85] Monreal-Escalante E., Bañuelos-Hernández B., Hernández M., Fragoso G., Garate T., Sciutto E., Rosales-Mendoza S. (2015). Expression of Multiple *Taenia Solium* Immunogens in Plant Cells Through a Ribosomal Skip Mechanism. Mol. Biotechnol..

[86] Jin S., Yang C., Huang J., Liu L., Zhang Y., Li S., Zhang L., Sun Q., Yang P. (2020). Conditioned medium derived from FGF-2-modified GMSCs enhances migration and angiogenesis of human umbilical vein endothelial cells. Stem Cell Res. Ther..

[87] Knoll A., Kankowski S., Schöllkopf S., Meier J.C., Seitz O. (2019). Chemo-biological mRNA imaging with single nucleotide specificity. Chem. Commun..

[88] Quenneville S., Labouèbe G., Basco D., Metref S., Viollet B., Foretz M., Thorens B. (2020). Hypoglycemia Sensing Neurons of the Ventromedial Hypothalamus Require AMPK-Induced Txn2 Expression But Are Dispensable For Physiological Counterregulation. Diabetes.

[89] Shibuta M.K., Matsuoka M., Matsunaga S. (2019). 2a Peptides Contribute To the Co-Expression of Proteins for Imaging and Genome Editing. Cytologia.

[90] Sinha D., Steyer B., Shahi P.K., Mueller K.P., Valiauga R., Edwards K.L., Bacig C., Steltzer S.S., Srinivasan S., Abdeen A. (2020). Human iPSC Modeling Reveals Mutation-Specific Responses to Gene Therapy in a Genotypically Diverse Dominant Maculopathy. Am. J. Hum. Genet..

[91] Kawano F., Okazaki R., Yazawa M., Sato M. (2016). A photoactivatable Cre-loxP recombination system for optogenetic genome engineering. Nat. Chem. Biol..

[92] Krishnamurthy V.V., Khamo J.S., Mei W., Turgeon A.J., Ashraf H.M., Mondal P., Patel D.B., Risner N., Cho E.E., Yang J. (2016). Reversible optogenetic control of kinase activity during differentiation and embryonic development. Development.

[93] Taslimi A., Zoltowski B., Miranda J.G., Pathak G.P., Hughes R.M., Tucker C.L. (2016). Optimized second-generation CRY2-CIB dimerizers and photoactivatable Cre recombinase. Nat. Chem. Biol..

[94] Luke G.A., Ryan M.D. (2018). Therapeutic applications of the ‘NPGP’ family of viral 2As. Rev. Med. Virol..

